# Complex approach for diagnosing acute respiratory distress syndrome in nosocomial pneumonia

**DOI:** 10.1186/cc14320

**Published:** 2015-03-16

**Authors:** A Kuzovlev, V Moroz, A Goloubev, S Polovnikov, V Stec

**Affiliations:** 1 V.A. Negovsky Research Institute of General Reanimatology, Moscow, Russia

## Introduction

Acute respiratory distress syndrome (ARDS) develops on the basis of nosocomial pneumonia (NP) in 30 to 35% of cases. A complex clinical approach is required for diagnosing it. The aim of this study was to investigate into the role of the PaO_2_/FiO_2_ ratio (P/F), extravascular lung water index (EVLWI) and surfactant protein D (SPD) as a complex diagnostic tool for ARDS in NP.

## Methods

The observational study in ICU ventilated septic patients with peritonitis (70%), pancreonecrosis (25%) and mediastinitis (5%) was done in 2010 and 2014. ARDS was diagnosed and staged according to the V.A. Negovsky Research Institute criteria and the Berlin definition. Plasma SPD was measured on ARDS diagnosis (day 0) and days 3 and 5 by the immunoenzyme essay (BioVendor, USA). Patients were treated according to the international guidelines. Data were statistically analyzed by STATISTICA 7.0, ANOVA and presented as median and 25 to 75th percentiles (ng/ml); *P *< 0.05 was considered statistically significant. Areas under the receiver operating curves were calculated.

## Results

Sixty-five patients (out of 450 screened) were enrolled in the study according to the inclusion/exclusion criteria. Patients were assigned into groups: NP + ARDS (*n *= 43, 43 ± 4.9 years old, M/F 39/4, mortality 23%) and NP (*n *= 22, 40 ± 5.1 years old, M/F 20/2, mortality 18%). Groups were comparable in APACHE II and SOFA scores on the baseline. In the NP + ARDS group SPD was higher at all points than in the NP group. Plasma SPD on day 0 >111.2 ng/ml yielded a sensitivity of 68.2% and specificity of 92.3% (AUC 0.85; 95% CI 0.684 to 0.945; *P *< 0.0001) for diagnosing ARDS in NP. P/F ratio on day 0 <280 yielded a sensitivity of 94.1% and specificity of 76.9% (AUC 0.89; 95% CI 0.744 to 0.952; *P *< 0.0001) and EVLWI on day 0 >8.3 ml/kg yielded a sensitivity of 94.1% and specificity of 92.3% (AUC 0.92; 95% CI 0.810 to 0.982; *P *< 0.0001) for the diagnosis of ARDS in NP. A complex ROC analysis (for SPD in the group of patients with P/F <280 and EVLWI >8.3) yielded a much better diagnostic accuracy of SPD: cutoff >93.7 ng/ml, sensitivity 81.0%, specificity 100.0% (AUC 0.96; 95% CI 0.817 to 0.998; *P *< 0.0001).

## Conclusion

A complex approach (P/F <280, EVLWI >8.3, SPD >93.7) presents as a sensitive and highly specific method for diagnosing ARDS in NP patients.

